# Clinical Findings Related to Temporomandibular Disorders in Schoolchildren Aged 4–16 Years: A Multicenter Cross-Sectional Study

**DOI:** 10.3390/jpm16080432

**Published:** 2026-08-15

**Authors:** Andrea Coello Hidalgo, Ana Alvear Miquilena, Domenica Yepez Zamora, Diego Quiguango Farias, Luis Chauca Bajaña, Maria Rodriguez Tates, Byron Velasquez Ron

**Affiliations:** 1 Carrera de Odontología, Universidad de Las Americas Ecuador (UDLA), Quito 170124, Ecuador; andrea.coello@udla.edu.ec (A.C.H.); ana.alvear@udla.edu.ec (A.A.M.); domenica.yepez.zamora@udla.edu.ec (D.Y.Z.); gaby.rodriguezt1@gmail.com (M.R.T.); 2 Carrera de Ciencias de La Salud, Universidad de Las Américas Ecaudor (UDLA), Quito 170124, Ecuador; diego.quiguango@udla.edu.ec; 3 School of Dentistry, Universidad Católica de Guayaquil (UCSG), Guayas 090101, Ecuador; luis.chuaca@ucsg.edu.ec

**Keywords:** temporomandibular disorders, schoolchildren, pediatric dentistry, Diagnostic Criteria for Temporomandibular Disorders, pain, logistic regression, cross-sectional study, personalized medicine, personalized dental care, precision oral health, risk stratification, early diagnosis

## Abstract

**Background**: Temporomandibular disorders (TMD) comprise a heterogeneous group of musculoskeletal conditions that may manifest during childhood and adolescence. However, standardized information regarding TMD-related clinical findings in pediatric populations remains limited because of methodological heterogeneity and the inconsistent application of validated diagnostic protocols. **Objective**: The aim of this study was to characterize clinical findings related to temporomandibular disorders in schoolchildren and to explore their potential contribution to individualized assessment strategies within the framework of personalized pediatric oral healthcare. **Methods**: A multicenter cross-sectional study was conducted in 1052 schoolchildren recruited from participating educational institutions. All participants underwent a standardized clinical examination that included assessment of masticatory muscle tenderness, temporomandibular joint tenderness, joint sounds, pain intensity, and perceived stress using an adapted pediatric DC/TMD protocol. Categorical variables were summarized as frequencies, percentages, and 95% confidence intervals (95% CIs). Associations between categorical variables were evaluated using Pearson’s chi-square test, and multivariable logistic regression was performed to identify factors independently associated with pain. **Results**: Pain during clinical examination was identified in 111 participants (10.6%; 95% CI: 8.8–12.6%), whereas temporomandibular joint sounds and myofascial pain were detected in 1.4% and 0.3% of participants, respectively. Most pain was classified as mild, and the masseter muscle was the most frequently affected anatomical site. Increasing age and female sex were independently associated with pain. Compared with children aged 4–6 years, participants aged 10–12 years (adjusted OR = 9.61; 95% CI: 2.22–41.51) and 13–16 years (adjusted OR = 35.49; 95% CI: 8.59–146.61) showed significantly greater odds of pain. Female participants also demonstrated higher odds of pain than males (adjusted OR = 7.49; 95% CI: 3.68–15.21; all *p* < 0.05). **Conclusions**: Clinically detectable TMD-related findings were generally uncommon among schoolchildren aged 4–16 years and were predominantly mild and muscular. Increasing age and female sex were independently associated with pain, emphasizing the importance of demographic factors in the early clinical expression of TMD. Standardized pediatric clinical examination protocols may facilitate early identification of TMD-related findings and support preventive strategies during childhood and adolescence. These findings support the implementation of individualized clinical assessment strategies for early identification of children at risk of temporomandibular disorders, contributing to personalized preventive and therapeutic approaches in pediatric oral healthcare.

## 1. Introduction

Temporomandibular disorders (TMD) comprise a heterogeneous group of musculoskeletal conditions affecting the temporomandibular joints, masticatory muscles, and associated structures [1]. They represent one of the leading causes of non-dental orofacial pain and functional impairment, frequently manifesting as pain, joint sounds, restricted mandibular movement, or altered mandibular function [2]. Although TMD has traditionally been regarded as a condition affecting adults, growing evidence indicates that its clinical manifestations may begin during childhood and adolescence, potentially influencing craniofacial growth, oral function, and quality of life if left unrecognized or untreated [3]. The etiology of TMD in pediatric populations is complex and multifactorial [4]. Biological, biomechanical, behavioral, and psychosocial factors—including parafunctional habits, trauma, occlusal characteristics, emotional stress, and genetic susceptibility—have all been implicated in the development of TMD-related clinical findings. However, the relative contribution of these factors remains uncertain because published studies differ substantially in their populations, diagnostic criteria, and clinical examination protocols [5]. The epidemiology of pediatric TMD remains difficult to interpret because of considerable methodological heterogeneity. Reported frequencies vary widely across studies owing to differences in sampling strategies, age ranges, examiner calibration, and diagnostic definitions [6]. Furthermore, many investigations rely primarily on self-reported symptoms or non-standardized examinations, which may underestimate or overestimate the true frequency of clinical findings in children. Consequently, comparisons among studies remain challenging, and the available evidence is often inconsistent [7]. The Diagnostic Criteria for Temporomandibular Disorders (DC/TMD) currently represents the international reference standard for clinical assessment of TMD in adults [8]. Although several components require adaptation for younger populations because of developmental and cognitive differences, standardized examination protocols based on the DC/TMD provide a more reliable framework for identifying TMD-related clinical findings in children and adolescents [9]. Such protocols improve diagnostic consistency and facilitate comparisons across studies while supporting early recognition of functional alterations before more severe clinical manifestations develop [10]. Despite increasing interest in pediatric TMD, relatively few large-scale clinical studies have characterized TMD-related findings using standardized examination protocols in school-aged children [11]. Most available reports focus on symptomatic patients or estimate disease prevalence, whereas fewer investigations describe the spectrum of clinical findings identified during routine clinical assessment in community-based pediatric populations [12]. Generating this information is clinically relevant because early recognition of functional or musculoskeletal alterations may facilitate timely preventive and conservative interventions [13]. Therefore, the aim of this multicenter cross-sectional study was to characterize temporomandibular disorder (TMD)-related clinical findings and to identify demographic factors associated with pain in schoolchildren aged 4–16 years using a standardized clinical examination based on an adapted DC/TMD protocol. Rather than estimating disease prevalence, this study sought to describe the frequency and distribution of TMD-related clinical findings identified during systematic clinical evaluation. Within the framework of personalized medicine, early recognition of clinical signs associated with temporomandibular disorders may facilitate individualized risk assessment and tailored preventive interventions. Rather than relying on uniform management strategies, personalized oral healthcare seeks to identify patient-specific characteristics—including age, clinical presentation, and functional findings—to optimize diagnosis and treatment planning. Consequently, describing the distribution of TMD-related clinical findings in pediatric populations may contribute to more individualized approaches to clinical decision-making.

## 2. Materials and Methods

### 2.1. Study Design

This multicenter cross-sectional study was conducted to characterize temporomandibular disorder (TMD)-related clinical findings in schoolchildren using a standardized clinical examination based on an adapted version of the Diagnostic Criteria for Temporomandibular Disorders (DC/TMD). The study was conducted from January 2024 to April 2026 and followed the recommendations of the Strengthening the Reporting of Observational Studies in Epidemiology (STROBE) Statement for cross-sectional studies.

Detailed school-level identifiers, examiner-specific participant assignments, characteristics of excluded participants, and documentation of the original sample-size calculation were not available in the archived study records and therefore could not be retrospectively reconstructed.

### 2.2. Study Population and Recruitment

Schoolchildren aged 4–16 years were recruited from participating educational institutions after authorization from school authorities. Fe Alegria Schools (Camal, Nayón, Luluncoto, Checa) Metropolitan District of Quito/Ecuador are present within projects to link society with the University. Parents or legal guardians received detailed information regarding the objectives and procedures of the study and provided written informed consent before participation. Verbal assent was also obtained from each child before the clinical examination [14].

**Figure 1:**
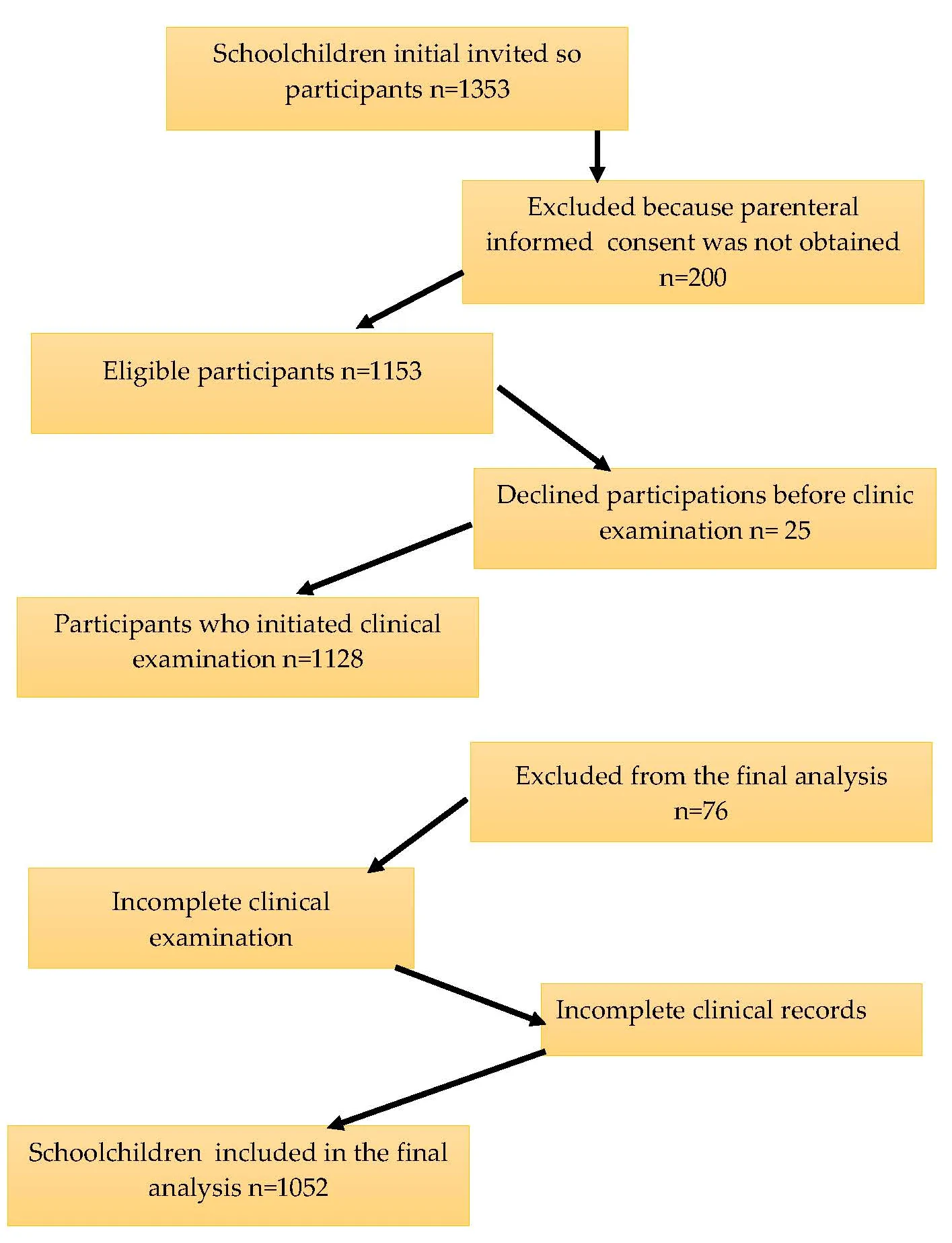
Flow diagram of participant recruitment, eligibility and inclusion in the final analysis.

A total of 1353 schoolchildren were initially invited to participate. Of these, 200 were excluded because parental consent was not obtained. The remaining 1153 children were considered eligible. Subsequently, 25 children declined participation before clinical examination, resulting in 1128 participants who initiated the study. During follow-up, 76 participants were excluded because of incomplete clinical examinations or incomplete records. Consequently, 1052 schoolchildren completed the standardized clinical assessment and were included in the final analysis (Figure 1).

### 2.3. Eligibility Criteria

Children were eligible if they

- were between 4 and 16 years of age;
- were enrolled in one of the participating schools;
- had written informed consent signed by a parent or legal guardian;
- provided verbal assent to participate;
- completed the standardized clinical examination;
- had complete clinical records available for analysis.

Children presenting systemic conditions or cognitive impairment preventing reliable clinical examination were also excluded. Participants were excluded if informed consent was not obtained, participation was withdrawn before completion of the clinical examination, or clinical records were incomplete.

### 2.4. Clinical Examination Protocol

Clinical examinations were performed by three calibrated examiners using a standardized protocol adapted from the Diagnostic Criteria for Temporomandibular Disorders (DC/TMD) for pediatric populations [9,15,16].

The examination protocol combined the standardized Axis I clinical assessment of the DC/TMD with a simplified pediatric adaptation of selected Axis II components. Clinical questions were reformulated into age-appropriate language to facilitate children’s understanding, while parents or legal guardians completed the corresponding home questionnaire to improve the accuracy and completeness of the collected information.

Before data collection, each participant underwent a structured clinical assessment that included the following:

#### Clinical Assessment Included

**Figure 2:**
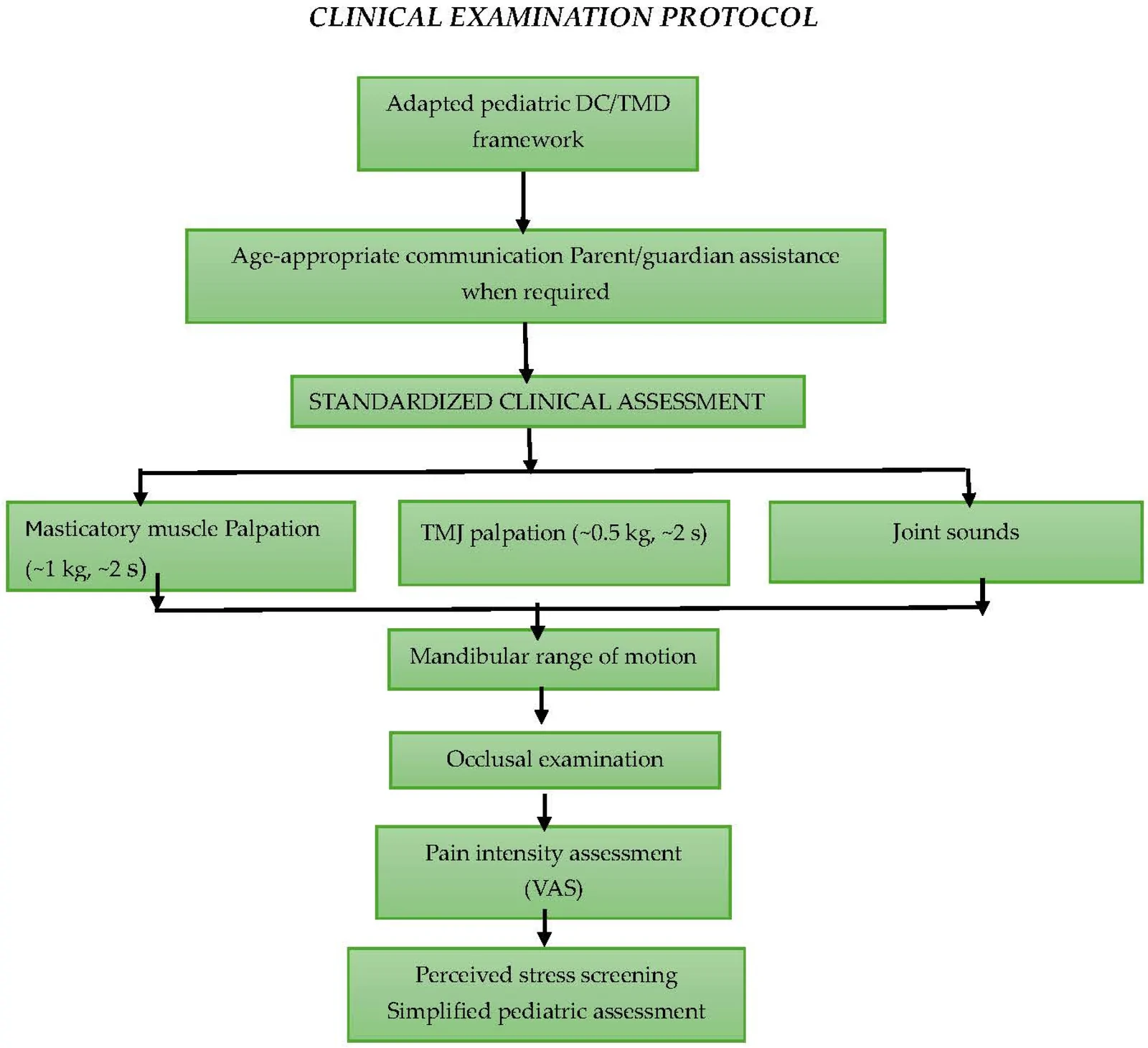
Standardized clinical examination protocol adapted from the DC/TMD framework for the pediatric study population. The assessment included age-appropriate communication, masticatory muscle and temporomandibular joint (TMJ) palpation, joint sound assessment, mandibular range of motion, occlusal examination, pain mapping, pain intensity assessment using a visual analog scale (VAS), and perceived stress screening. Parent or legal guardian assistance was provided when required to facilitate comprehension.

- Muscle palpation;
- TMJ palpation;
- Joint sounds;
- Mandibular range of motion;
- Occlusal examination;
- Pain mapping;
- VAS;
- Stress questionnaire (Figure 2).

The clinical protocol was adapted to the developmental characteristics of the participants by using age-appropriate language and, when necessary, parent- or guardian-assisted responses. Because the study was conducted using a simplified clinical record developed for field assessment, some historical methodological details regarding the specific age-dependent adaptations and administration procedures could not be reliably reconstructed from the original study documentation. Accordingly, no additional assumptions were introduced retrospectively.

### 2.5. Examiner Calibration

Before participant recruitment, the three clinical examiners completed a 1-month calibration and training program conducted by the principal investigator (B.V.), who has extensive clinical experience in the diagnosis and management of temporomandibular disorders. The training consisted of theoretical instruction and supervised practical sessions focused on the standardized application of the adapted pediatric Diagnostic Criteria for Temporomandibular Disorders (DC/TMD) protocol [9,15].

Calibration emphasized the standardization of muscle and temporomandibular joint palpation, assessment of mandibular movements, identification of joint sounds, pain recording procedures, and completion of the simplified clinical record adapted from the DC/TMD framework [17,18]. Particular attention was given to achieving consistency in the interpretation and recording of clinical findings among the three examiners.

Inter-examiner agreement was assessed using Cohen’s kappa coefficient, yielding an overall value of κ = 0.87. According to the conventional interpretation proposed by Landis and Koch [19], this value indicates strong agreement. However, because the original calibration dataset did not preserve variable-specific or examiner-pair-specific agreement estimates, this overall coefficient should be interpreted as a general indicator of agreement rather than definitive evidence of reproducibility across all clinical variables and all three examiners [20,21,22].

### 2.6. Study Variables

Primary outcome:

Presence of pain during standardized clinical examination.

Secondary outcomes:

- Myofascial pain;
- TMJ sounds;
- Pain location;
- Pain intensity;
- Stress.

Independent variables:

- Age;
- Sex.

### 2.7. Statistical Analysis

Statistical analyses were performed using IBM SPSS Statistics version 31 (IBM Corp., Armonk, NY, USA). Categorical variables were summarized as frequencies, percentages, and 95% confidence intervals (95% CIs) estimated using the Wilson score method. Associations between categorical variables were evaluated using Pearson’s chi-square test. Variables significantly associated with pain were entered into a multivariable logistic regression model to estimate adjusted odds ratios (ORs) and corresponding 95% confidence intervals. A two-sided *p*-value < 0.05 was considered statistically significant.

### 2.8. Ethical Considerations

The study was conducted in accordance with the ethical principles established in the Declaration of Helsinki.

The research protocol was reviewed and approved by the Research Ethics Committee of Universidad de Las Américas (Approval No. CBE1705901/2024; 4 January 2024).

Written informed consent was obtained from all parents or legal guardians before enrollment, and verbal assent was obtained from each participating child. Participant confidentiality was preserved by anonymizing all clinical records before statistical analysis.

## 3. Results

### 3.1. Participant Characteristics

A total of 1052 schoolchildren were included in the study. Females represented 64.4% of the sample (678/1052; 95% CI: 61.5–67.3%), whereas males accounted for 35.6% (374/1052; 95% CI: 32.7–38.5%). The largest proportion of participants belonged to the 13–16-year age group (33.5%), followed by those aged 10–12 years (25.3%), 7–9 years (21.8%), and 4–6 years (19.5%). Detailed demographic characteristics are presented in Table 1.

### 3.2. Clinical Findings

Clinical examination identified pain in 111 participants (10.6%; 95% CI: 8.8–12.6%). Mild pain was the most frequent finding (6.6%), followed by moderate (3.2%) and severe pain (0.8%). Only three participants (0.3%) fulfilled the criteria for myofascial pain, while temporomandibular joint sounds were detected in 15 participants (1.4%).

Most children exhibited normal stress levels, whereas only 29 participants (2.8%) presented mild, moderate, or moderately severe stress. A complete summary of the clinical findings is presented in Table 2.

### 3.3. Anatomical Distribution of Tenderness

The anatomical location of tenderness was recorded in 98 of the 111 participants presenting pain. The masseter muscle was the most frequently affected structure, being involved in all recorded cases (100.0%; 95% CI: 96.2–100.0%). Tenderness of the temporal muscle was identified in 46.9% of participants, followed by temporomandibular joint tenderness (41.8%) and tenderness involving other masticatory muscles (7.1%).

Because tenderness could be identified at more than one anatomical site in the same participant, percentages exceeded 100%. The anatomical distribution of tenderness is summarized in Table 3.

### 3.4. Factors Associated with Pain

The bivariate analysis demonstrated statistically significant associations between the presence of pain and both sex and age group (Pearson’s χ^2^, *p* < 0.001 for both comparisons). Overall, female sex and increasing age were independently associated with a higher likelihood of pain during clinical examination.

The distribution of pain according to sex and age group is presented in Table 4.

After adjustment in a multivariable logistic regression model, female participants were significantly more likely to present pain than males (adjusted OR = 7.49; 95% CI: 3.68–15.21; *p* < 0.001).

Age was also independently associated with pain. Compared with children aged 4–6 years, those aged 10–12 years exhibited significantly greater odds of pain (adjusted OR = 9.61; 95% CI: 2.22–41.51; *p* = 0.002), whereas participants aged 13–16 years showed the strongest association (adjusted OR = 35.49; 95% CI: 8.59–146.61; *p* < 0.001). No statistically significant difference was observed for children aged 7–9 years (adjusted OR = 1.66; 95% CI: 0.27–10.07; *p* = 0.583). The results of the regression analysis are presented in Table 5.

## 4. Discussion

The present multicenter cross-sectional study characterized temporomandibular disorder (TMD)-related clinical findings in 1052 schoolchildren aged 4–16 years using a standardized clinical examination adapted from the Diagnostic Criteria for Temporomandibular Disorders (DC/TMD) [9,15,16,23,24]. From the perspective of personalized medicine, the present findings emphasize that children should not be considered a homogeneous population with respect to temporomandibular health. The variability observed in clinical manifestations suggests that individualized clinical assessment may allow clinicians to the variability observed in clinical manifestations suggests that individualized clinical assessment may help clinicians recognize children presenting demographic or clinical characteristics associated with currently observed TMD-related findings, thereby supporting more tailored clinical evaluation and follow-up. Three principal findings emerged from this investigation. First, clinically relevant TMD-related findings were uncommon in the overall study population. Second, when abnormalities were identified, muscular tenderness predominated over temporomandibular joint involvement [25,26]. Third, increasing age and female sex were independently associated with the presence of pain, highlighting demographic characteristics that may influence the early clinical expression of TMD-related conditions. The relatively low frequency of pain observed in the present study is consistent with previous investigations reporting that clinically significant TMD-related pain is less common in children than in adults [27]. Although the reported prevalence of pediatric TMD varies substantially because of differences in study populations, diagnostic criteria, and clinical examination protocols, community-based investigations generally report predominantly mild clinical manifestations [28]. Developmental factors, lower cumulative functional loading of the masticatory system, and the ongoing maturation of craniofacial structures may partially explain these findings. Furthermore, children may have greater difficulty recognizing and accurately describing pain than adolescents or adults, potentially influencing symptom reporting [14,29]. Muscular tenderness represented the predominant clinical finding, whereas temporomandibular joint involvement and joint sounds were identified in only a small proportion of participants. Similar observations have been reported in previous pediatric studies, suggesting that muscular alterations frequently precede intra-articular disorders during growth and development [30]. Functional overload, parafunctional habits, and adaptive neuromuscular responses have all been proposed as mechanisms contributing to these early clinical manifestations [31]. Therefore, muscular tenderness identified during childhood should be considered a relevant clinical finding that deserves monitoring, even in the absence of overt joint pathology. One of the most important findings of the present investigation was the independent association between age and pain. After adjustment for sex, participants aged 10–12 years and particularly those aged 13–16 years demonstrated significantly greater odds of presenting pain than younger children. Although the adjusted analysis showed progressively higher odds of pain across older age groups, these estimates should be interpreted cautiously. The wide confidence intervals, particularly for the older age categories relative to the youngest reference group, reflect the small number of pain-positive participants in the younger age groups and indicate substantial uncertainty in the magnitude of the estimated associations. Therefore, the direction of the association appears consistent, whereas the precise effect sizes should not be overinterpreted. Although the present cross-sectional design does not permit causal inference, these findings agree with epidemiological studies reporting an increasing frequency of TMD signs and symptoms during late childhood and adolescence [32]. Craniofacial growth, hormonal changes associated with puberty, progressive functional loading, and increasing exposure to parafunctional behaviors may all contribute to this age-related pattern [33]. These findings reinforce the importance of individualized diagnostic pathways in pediatric dentistry, where treatment decisions should be based on each child’s specific clinical characteristics rather than on generalized assumptions regarding age or symptom presentation. This finding suggests that preventive screening strategies may be particularly relevant during early adolescence, especially among girls. This observation is consistent with previous reports describing a higher prevalence of TMD-related pain among females beginning during adolescence [34]. Proposed explanations include hormonal influences, differences in pain perception, psychosocial factors, and sex-related variations in neuromuscular regulation. Although these mechanisms were not directly evaluated in the present study, the persistence of the association after adjustment suggests that sex should be considered an important demographic factor during pediatric TMD assessment. Most participants exhibited normal or apparently normal perceived stress levels, and only a small proportion presented clinically relevant stress. Although psychosocial factors are recognized as important components of the biopsychosocial model of TMD [35], this finding should be interpreted cautiously because stress was assessed using a simplified clinical instrument rather than comprehensive pediatric psychological questionnaires. Future longitudinal studies incorporating validated psychosocial instruments may better clarify the contribution of emotional factors to early TMD manifestations [36,37]. Several strengths should be acknowledged. A major strength of this study is the large multicenter cohort of 1052 schoolchildren clinically evaluated using a standardized protocol adapted from the DC/TMD framework. The available calibration assessment yielded an overall κ value of 0.87, indicating strong agreement; however, this finding should be interpreted in light of the absence of variable-specific and examiner-pair-specific reliability estimates of the clinical examination procedures. In addition, the incorporation of confidence intervals, inferential statistical analyses, and multivariable logistic regression strengthened the interpretation of the findings beyond descriptive epidemiology.

Nevertheless, several limitations should be considered. The cross-sectional design precludes establishing causal relationships between demographic characteristics and TMD-related findings. Participants were recruited from selected educational institutions rather than through probability-based sampling, which may limit the external validity of the results. Furthermore, although a standardized pediatric protocol adapted from the DC/TMD was employed, imaging examinations such as magnetic resonance imaging or cone-beam computed tomography were not performed; therefore, subclinical intra-articular alterations could not be confirmed [38]. Finally, psychosocial assessment was limited to a simplified clinical protocol and therefore may not fully capture the complex biopsychosocial mechanisms underlying pediatric TMD, highlighting the importance of comprehensive multidimensional assessment in future studies [39]. Overall, the present findings indicate that clinically detectable TMD-related alterations are generally uncommon among schoolchildren; however, their frequency increases with age and is significantly higher among females. These findings support the implementation of standardized pediatric clinical examination protocols for the early identification of children at greater risk of TMD and may contribute to individualized preventive strategies and personalized clinical decision-making during childhood and adolescence [40]. These findings contribute to the growing body of evidence supporting standardized pediatric TMD assessment and may facilitate earlier identification of children at increased risk of developing clinically significant temporomandibular disorders.

## 5. Conclusions

This multicenter cross-sectional study showed that clinically detectable TMD-related findings were generally uncommon among schoolchildren aged 4–16 years evaluated using a standardized clinical examination adapted from the DC/TMD framework. When present, clinical findings were predominantly muscular and generally mild. Increasing age and female sex were independently associated with the presence of pain, indicating that demographic characteristics may contribute to differences in the clinical expression of TMD-related findings during childhood and adolescence. Standardized pediatric assessment may therefore provide useful information for individualized clinical evaluation. However, because of the cross-sectional design, these findings do not establish future TMD risk or the effectiveness of preventive interventions. Longitudinal studies are needed to determine the prognostic relevance of these associations and their potential contribution to personalized pediatric oral healthcare.

## 6. Clinical Implications

The present findings support the value of standardized TMD assessment as part of pediatric clinical examination. Older children and adolescents, particularly females, showed a higher frequency of pain-related findings in this sample and may therefore warrant particular attention during clinical assessment. These cross-sectional associations may inform individualized clinical evaluation but should not be interpreted as predictors of future TMD or as evidence supporting specific preventive interventions. Prospective longitudinal studies are required to determine whether these demographic and clinical patterns can contribute to risk stratification, monitoring strategies, or personalized preventive approaches.

## 7. Study Limitations

The principal limitations of this study include its cross-sectional design, which precludes causal or prognostic inference, and the recruitment of participants from selected educational institutions, which may limit the generalizability of the findings. Although an overall inter-examiner agreement of κ = 0.87 was obtained during calibration, variable-specific and examiner-pair-specific reliability estimates were not available in the archived calibration records; therefore, this coefficient should be interpreted cautiously as a general measure of agreement rather than definitive evidence of reproducibility across all examination components. Some variables also depended on children’s subjective responses and may have been influenced by developmental differences in pain perception and communication abilities. In addition, the small number of pain-positive participants in the younger age groups resulted in wide confidence intervals for some age-related estimates, limiting the precision of the corresponding effect sizes. Imaging examinations were not performed; therefore, subclinical temporomandibular joint alterations could not be confirmed. Finally, some methodological details of the original field protocol could not be retrospectively reconstructed from the archived study documentation. Future longitudinal multicenter studies incorporating imaging techniques, validated pediatric psychosocial instruments, repeated clinical assessments, and detailed examiner-specific reliability analyses are needed to clarify the natural history and prognostic significance of TMD-related findings throughout childhood and adolescence.

## Figures and Tables

**Table 1:** Participant characteristics.

Characteristic	*n*	%	95% CI
Total	1052	100.00	95 CI
Female	678	64.45	61.5–67.3
Male	374	35.55	32.7–38.5
Age 4–6	205	19.49	17.2–22.0
Age 7–9	229	21.77	19.4–24.4
Age 10–12	266	25.29	22.8–28.0
Age 13–16	352	33.46	30.7–36.4

Abbreviations: CI, confidence interval. Data are presented as *n* (%). Percentages were calculated using the total study population (N = 1052). Ninety-five percent confidence intervals (95% CIs) were estimated using the Wilson score method.

**Table 2:** Recorded clinical variables.

Finding	*n*	%	95% CI
Any coded pain	111	10.55	8.84–12.55
Mild pain	69	6.56	5.22–8.22
Moderate pain	34	3.23	2.32–4.48
Severe pain	8	0.76	0.39–1.49
Recorded myofascial pain	3	0.29	0.10–0.84
TMJ sounds	15	1.43	0.87–2.34
Elevated stress (codes 3–5)	29	2.76	1.93–3.93
Location available among pain-positive	98	88.29	80.99–93.03
Location missing among pain-positive	13	11.71	6.97–19.0

Abbreviations: CI, confidence interval; TMJ, temporomandibular joint. Data are presented as *n* (%). Percentages were calculated using the total study population (N = 1052). Ninety-five percent confidence intervals (95% CIs) for proportions were estimated using the Wilson score method.

**Table 3:** Anatomical distribution of muscle and temporomandibular joint tenderness.

Localización Anatómica	*n*	%	95% CI
Temporal muscles	46	46.94	37.36–56.75
Masseter muscles	98	100.00	96.23–100.00
Other muscles	7	7.14	3.50–14.02
Temporomandibular joint (TMJ)	41	41.84	32.56–51.73

Abbreviations: CI, confidence interval; TMJ, temporomandibular joint. Data are presented as *n* (%). Participants could present tenderness in more than one anatomical location; therefore, percentages do not sum to 100%. Percentages and 95% confidence intervals (95% CIs) were calculated using the 98 participants with recorded anatomical location as the denominator. Confidence intervals were estimated using the Wilson score method.

**Table 4:** Association between participant characteristics and the presence of pain.

Variable	Pain *n* (%)	No Pain *n* (%)	Total	*p* Value
Sex				
Female	102 (15.0)	576 (85.0)	678	<0.001
Male	9 (2.4)	365 (97.6)	374	
Age group (years)				
4–6	2 (1.0)	203 (99.0)	205	
7–9	3 (1.3)	226 (98.7)	229	
10–12	22 (8.3)	244 (91.7)	266	
13–16	84 (23.9)	268 (76.1)	352	<0.001

Data are presented as *n* (%). Percentages were calculated within each category of the independent variable. *p* Values were obtained using Pearson’s chi-square test. Statistical significance was established at *p* < 0.05.

**Table 5:** Multivariable logistic regression analysis for factors associated with pain.

Variable	Adjusted OR	95% CI	*p* Value
Sex			
Female vs. Male	7.49	3.68–15.21	<0.001
Age group (years)			
7–9 vs. 4–6	1.66	0.27–10.07	0.583
10–12 vs. 4–6	9.61	2.22–41.51	0.002
13–16 vs. 4–6	35.49	8.59–146.61	<0.001

Abbreviations: OR, odds ratio; CI, confidence interval. Adjusted odds ratios (ORs) and 95% confidence intervals (95% CIs) were estimated using multivariable logistic regression. The model included sex and age group simultaneously. Male sex and the 4–6-year age group were used as the reference categories. Statistical significance was established at *p* < 0.05.

## Data Availability

Available in Zenodo: DOI 10.5281/zenodo.21329054.

## Abbreviations

The following abbreviations are used in this manuscript:

DC/TMD	Diagnostic Criteria for Temporomandibular Disorders
TMJ	Temporomandibular Joint
TMD	Temporomandibular Disorder
